# The Association of Vitamin D, Growth/Differentiation Factor 5 (GDF-5) Gene Polymorphism, and Serum GDF-5 Protein in Obese Patients With Knee Osteoarthritis

**DOI:** 10.7759/cureus.48350

**Published:** 2023-11-06

**Authors:** Abdulraheem Almalki, Amal F Gharib, Mazen Almehmadi, Afaf Alharthi, Ohud Alsalmi, Alaa H Alsulimani, Rasha H Alanazi, Ahmed A AlWthenani, Maeidh Alotaibi, Fawaz T AlZaidi

**Affiliations:** 1 Department of Clinical Laboratory Sciences, College of Applied Medical Sciences, Taif University, Taif, SAU; 2 Genetics, King Faisal Medical Complex (KFMC) And Research Center, Taif, SAU; 3 Genetics, Enayat Al-Aufaq Medical Complex, Tabuk, SAU; 4 Hospital-Based Medicine, King Faisal Medical Complex (KFMC) and Research Center, Taif, SAU; 5 Health Affairs, Ministry of Health, Taif, SAU

**Keywords:** obese, pcr-rflp, gdf-5 protein, vitamin d, gdf-5 gene polymorphism, knee osteoarthritis

## Abstract

Background and objective

Osteoarthritis (OA) is influenced by genetics and environmental factors, including vitamin D deficiency. This study aimed to investigate the association between vitamin D levels, growth/differentiation factor 5 (GDF-5) gene polymorphism, and serum GDF-5 in obese females with knee OA (KOA) in Saudi Arabia.

Methodology

The study enrolled 60 female patients with OA and 60 healthy females as controls. Blood samples were collected to evaluate the GDF-5 T>C (rs143383) polymorphism using polymerase chain reaction-restriction fragment length polymorphism (PCR-RFLP). The study also measured serum levels of vitamin D, GDF-5, calcium, uric acid, lipid profiles, C-reactive protein (CRP), and erythrocyte sedimentation rate (ESR), and assessed the participants' BMI.

Results

The study demonstrated that KOA patients had reduced vitamin D levels in their bodies, along with GDF-5 and calcium. However, they had increased levels of uric acid, lipid profile, CRP, and ESR. Strong correlations were observed between vitamin D levels, lipid profile, CRP, ESR, BMI, GDF-5 gene polymorphisms, and GDF-5 protein. Genotype analysis showed KOA patients had TT (30%), TC (50%), and CC (20%) genotypes, while the control group showed TT (22%), TC (35%), and CC (43%) genotypes. Allele analysis revealed a noteworthy association between the T allele and KOA; the C allele was more common in the control group.

Conclusions

The study findings provide valuable insights into the association of vitamin D levels with GDF-5 T>C (rs143383) polymorphism, GDF-5 protein, and inflammatory markers in obese Saudi females with KOA. These findings suggest potential associations between these biomarkers and the pathogenesis or progression of KOA.

## Introduction

Osteoarthritis (OA) is a widespread condition that significantly impacts the quality of life among those affected by it. Although it can affect various joints, it mainly impacts the hips and knees, which are crucial joints that bear most of the body's weight [1]. This condition is highly complex and entails the progressive degradation of cartilage, bone remodeling, and a limited inflammatory reaction within the affected region [2]. A hallmark of this disease is the gradual breakdown of joint cartilage caused by excessive destructive processes that outweigh the body's ability to regenerate tissue. To restore joint function and promote cartilage growth, it may be necessary to pursue medical treatment options [3].

Knee osteoarthritis (KOA) causes changes in knee function, resulting in social and economic consequences, as well as symptoms associated with the knee joint [4]. It is associated with a serious economic burden on healthcare resources in many nations and is currently considered a significant societal issue [2]. Healthcare costs can be directly linked to various aspects of KOA, such as the need for joint replacements, or can be greatly affected by the use of medication [5]. OA linked to obesity is a significant health issue that can greatly affect a person's ability to move around and their overall well-being. This illness affects a substantial portion of individuals who are overweight, with more than half of those with advanced KOA requiring a total knee replacement also being obese [6]. The leading factor contributing to obesity-related OA is the mechanical constraints resulting from the excessive and intense pressure placed on the joints and the concurrent misalignment of the joints and weakened muscles [7].

Maintaining sufficient vitamin D levels is essential to ensure optimal bone health. This vital nutrient is responsible for proper skeleton and joint regeneration and calcium and phosphorus metabolism. Insufficient levels of vitamin D are linked with numerous problems, such as decreased osteoblastic performance, reduced bone density, and impaired osteochondral renewal [8]. Based on the literature, vitamin D deficiency could be integral to the progress of KOA [9]. As people age, their vitamin D reservoir decreases, leading to subclinical vitamin D deficiency among older individuals [10]. Vitamin D receptors have been discovered in human chondrocytes. This vitamin has a significant impact on cartilage through these receptors, as it can stimulate the production of proteoglycan in mature chondrocytes [11]. Insufficient vitamin D levels can negatively affect bone metabolism and potentially contribute to the development of OA through various mechanisms [9]. On the other hand, vitamin D can potentially inhibit the progression of OA by promoting the formation of new bone tissue and reducing the dysfunctional processes involved [12].

Growth/differentiation factor 5 (GDF-5) is a natural growth factor that is necessary for creating fetal cartilage and maintaining adult cartilage [13]. Several studies have shown that GDF-5 can enhance collagen production in human chondrocytes [14,15], by reducing the expression of matrix metallopeptidase (MMP13) and A disintegrin and metalloproteinase with thrombospondin motifs (ADAMTS4). GDF-5 regulates soft bone formation in fractures and aids in producing new cartilage to meet the hyaline demands of affected joints [15]. Moreover, GDF-5 has displayed the capability to boost cartilage generation by mesenchymal stem cells (MSC). The effect of GDF-5 polymorphism in OA is still being researched. One of the most studied GDF-5 polymorphisms concerning OA is the rs143383 single nucleotide polymorphism (SNP) located in the gene's promoter region. SNPs in the promoter region impact gene expression by influencing promoter activity, transcription factor binding, DNA methylation, and histone changes. These variants may lead to decreased GDF-5 expression or activity, which could potentially affect joint development and cartilage maintenance, thereby contributing to the development of OA [16].

This study aims to explore the relationship between serum vitamin D levels, GDF-5 T>C (rs143383) polymorphism, and serum GDF-5 in obese Saudi patients with KOA.

## Materials and methods

Between October 2022 and April 2023, a case-control study was conducted at the College of Applied Medical Sciences, Taif University. For this study, a total of 120 Saudi subjects were enrolled and classified into two subgroups. The first group consisted of 60 females aged 25-65 years who had both obesity and KOA. These individuals were receiving treatment at the Rheumatology Clinic of the King Faisal Medical Complex in Taif. The second group, serving as the control group, comprised 60 healthy individuals who were carefully matched with the first group in terms of age and gender. The control group participants did not display any symptoms of OA or other medical conditions. Prior to the commencement of the study, all participants provided written consent, indicating their voluntary participation. The research protocol received approval from the Scientific Research Ethics Committee of the King Faisal Medical Complex in Taif (no. 2023-B-1).

The patients were diagnosed with KOA based on the classification criteria of the American College of Rheumatology. For the confirmation of the diagnosis, anteroposterior and lateral views of knee radiographs were taken [17]. Additionally, the Kellgren and Lawrence scoring system was used to evaluate functional disability associated with OA in all patients [18]. The severity of symptoms of the KOA was assessed using the Western Ontario and McMaster Universities Osteoarthritis Index (WOMAC) [19]. The BMI was calculated for each participant using the formula BMI = weight (kg)/height (m^2^) [20].

The study excluded patients with other autoimmune diseases, such as systemic lupus erythematosus, mixed connective tissue diseases, scleroderma, and dermatomyositis. Also, those with non-autoimmune conditions like fibromyalgia, hypermobility, and hereditary connective tissue disease were also excluded. Furthermore, subjects who had taken vitamin D and calcium supplements within three months prior were excluded.

Sampling and biochemical analysis

Each subject underwent a clean venipuncture technique to provide a 10 ml fasting blood sample. The sample was split into two parts; 5 ml was mixed with an anticoagulant (EDTA) for DNA extraction, C-reactive protein (CRP), and erythrocyte sedimentation rate (ESR) determination. The remaining sample was allowed to coagulate and then centrifuged to obtain serum, which was stored at -20 °C for testing levels of vitamin D, GDF-5, calcium, uric acid, and lipid profile.

We used Cobas 6000 (e601) electrochemiluminescence (ECL) technology to measure the vitamin D serum levels in all samples. We used a human GDF-5 competitive ELISA kit from My Biosource (MyBioSource, Inc., Vancouver, Canada) to estimate the GDF-5 protein. Additionally, the analysis of calcium, uric acid, and lipid profiles (cholesterol, triglycerides, HDL-C, and LDL-C) was performed using Cobas 6000 (c501) photometric technology. CRP levels were assessed using the Beckman Coulter IMMAGE® 800 Protein Chemistry Analyzer (Beckman Coulter, Inc., Brea, CA). Furthermore, the ESR was determined using Alifax® technology.

Genotyping of GDF-5 gene polymorphism (rs143383, T>C)

The genomic DNA was extracted and purified from peripheral blood samples of patients with KOA as well as controls. The QIAamp DNA Mini Kit from Qiagen in California was used for this process. After extraction, the DNA was kept at -80 °C until it was analyzed for genotyping.

Detection of GDF-5 promoter polymorphism (rs143383, T>C) using PCR-RFLP

The genotyping of the GDF-5 T>C polymorphism was carried out using the polymerase chain reaction-restriction fragment length polymorphism (PCR-RFLP). A 25 µl reaction volume was used for the PCR amplification, employing recombinant Taq polymerase master mix from Thermo Fisher Scientific Ballics UAB (Vilnius, Lithuania). The primer sequences utilized to amplify the promoter region (rs143383) of the GDF-5 gene were as follows: forward: 5′-GATTTTTTCTGAGCACCTGCAGG-3′; reverse: 5′-GTGTGTGTTTGTATCCAG -3′.

The amplification process started with an initial denaturation step at 95 °C for five minutes. This was followed by 35 cycles, each consisting of denaturation at 94 °C for one minute, annealing at 58 °C for one minute, and extension at 72 °C for one minute. Finally, a final extension step was performed at 72 °C for 10 minutes. Subsequently, the PCR product (10 µL) was subjected to a four-hour incubation with BsiEI restriction enzyme (three units) at 37 °C. The resulting digested fragments were separated on a 2% agarose gel stained with ethidium bromide and visualized using a UV transilluminator. The observed fragment lengths corresponded to 104 and 230 bp for the CC genotype; 104, 230, and 344 bp for the TC genotype; and 344 bp for the TT genotype.

Statistical analysis

The analysis of our data was conducted using GraphPad Prism version 8. To compare different variables between the studied groups, we employed the student t-test analysis for parametric data. This statistical test allowed us to determine significant differences between the groups. Additionally, we used Pearson's correlation coefficient to assess the association between vitamin D and other parameters examined in the studied groups. Furthermore, the chi-square (X^2^) test was utilized to investigate the relationship between two qualitative variables. A p-value of less than 0.05 within a 95% confidence interval was considered statistically significant.

## Results

The study involved 60 individuals diagnosed with KOA and 60 healthy controls. The patients' ages ranged from 25 to 65 years, with a mean age of 53.12 ± 10.87 years. In comparison, the control subjects' ages ranged from 27 to 67 years, with a mean age of 55.16 ± 11.34 years.

Analysis of biochemical parameters in all participants

Table 1 provides a comprehensive comparison of various laboratory parameters between KOA patients and control subjects. The following parameters were examined: serum vitamin D, GDF-5 protein, calcium, cholesterol, triglycerides, HDL-C, LDL-C, uric acid, ESR, CRP, and BMI. The analysis revealed notable differences between the two groups. The KOA group exhibited lower mean levels of vitamin D (23.94 ± 10.99 ng/mL), GDF-5 protein (11.09 ± 2.753 ng/mL), and calcium (8.54 ± 1.23 mg/dL) as compared to the control group. Conversely, the KOA group displayed higher mean levels of cholesterol (234.4 ± 51.63 mg/dL), triglycerides (225.3 ± 31.58 mg/dL), LDL-C (194.3 ± 38.37 mg/dL), uric acid (8.79 ± 1.53 mg/dL), ESR (29.62 ± 12.11 mm/hr), CRP (20.62 ± 10.75 mg/L), and BMI (48.94 ± 11.22 kg/m^2^). Additionally, the KOA group had a lower mean HDL-C (37.46 ± 9.24 mg/dL) in contrast to the higher mean HDL-C (67.70 ± 10.65 mg/dL) observed in the control group.

**Table 1:** A comparative analysis of biochemical parameters between patients and controls KOA: knee osteoarthritis; SD: standard deviation; GDF-5: growth/differentiation factor 5; HDL-C: high-density lipoprotein cholesterol; LDL-C: low-density lipoprotein cholesterol; ESR: erythrocyte sedimentation rate; CRP: C-reactive protein; BMI: body mass index

Biochemical parameter	KOA (60)	Controls (60)	t-test	P-value
	Mean ± SD			
Vitamin D, ng/mL	23.94 ± 10.99	38.20 ± 6.081	8.791	<0.0001
GDF-5 protein, ng/mL	11.09 ± 2.753	15.03 ± 3.426	6.957	<0.0001
Calcium, mg/dL	8.54 ± 1.23	10.15 ± 1.19	3.084	<0.0001
Cholesterol, mg/dL	234.4 ± 51.63	150.2 ± 42.78	9.727	<0.0001
Triglycerides, mg/dL	225.3 ± 31.58	148.1 ± 48.09	10.39	<0.0001
HDL-C, mg/dL	37.46 ± 9.24	67.70 ± 10.65	16.60	<0.0001
LDL-C, mg/dL	194.3 ± 38.37	72.80 ± 11.01	23.57	<0.0001
Uric acid, mg/dL	8.79 ± 1.53	5.196 ± 1.99	11.08	<0.0001
ESR, mm/hr	29.62 ± 12.11	9.33 ± 1.37	11.16	<0.0001
CRP, mg/L	20.62 ± 10.75	5.55 ± 2.22	7.995	<0.0001
BMI, kg/m^2^	48.94 ± 11.22	24.52 ± 8.8	13.26	<0.0001

Correlation between vitamin D levels and BMI 

**Figure 1:**
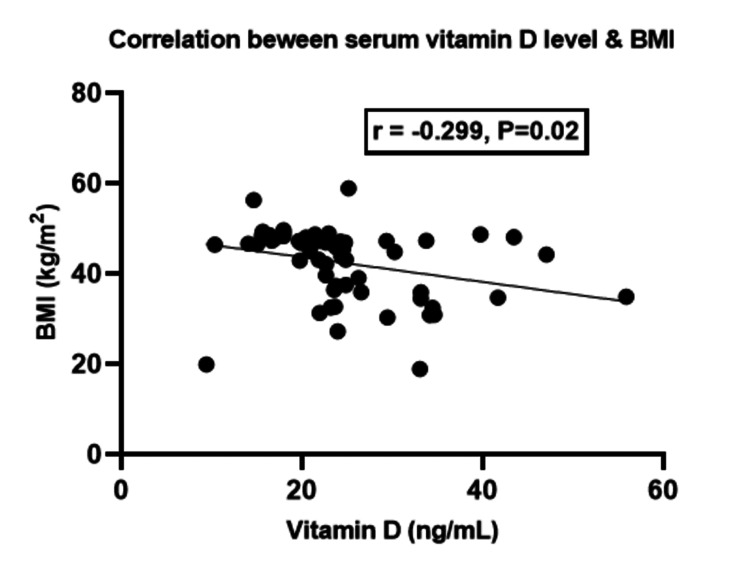
Correlation of vitamin D levels with body mass index in knee osteoarthritis patients

The regression analysis showed a notable inverse relationship between vitamin D levels and BMI (r = -0.299, p = 0.02) (Figure 1).

Correlation between vitamin D levels and GDF-5 protein

**Figure 2:**
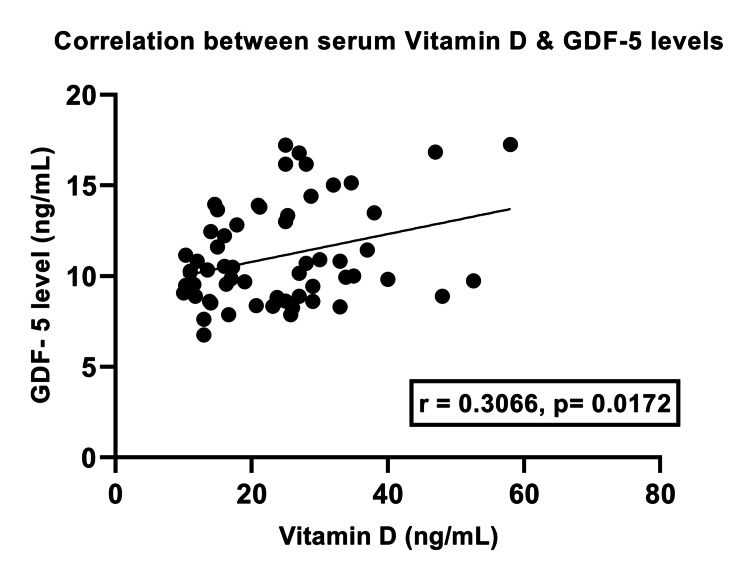
Correlation of serum vitamin D with growth/differentiation factor 5 protein in knee osteoarthritis patients

Our analysis showed that there was a statistically significant positive correlation between the levels of serum vitamin D and GDF-5 protein in KOA patients (r = 0.3066, p = 0.0172) (Figure 2).

Correlation between serum vitamin D, calcium, and uric acid levels

**Figure 3:**
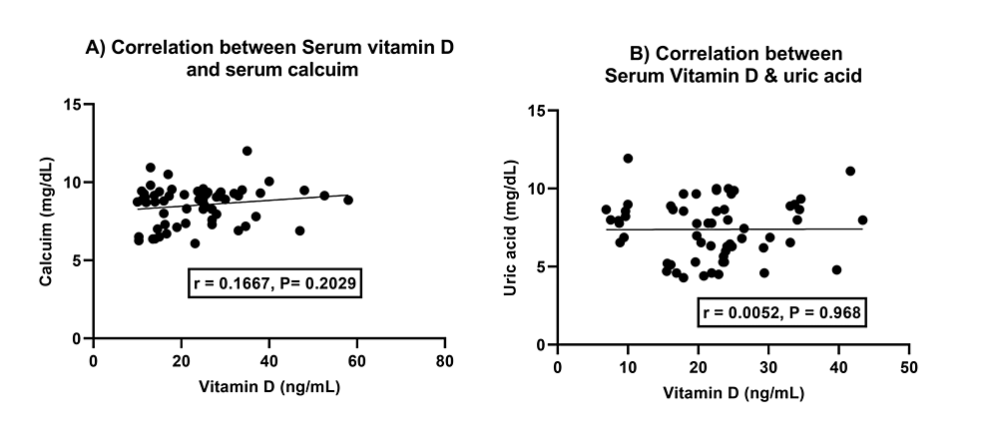
Association between vitamin D and calcium (A) and vitamin D and uric acid levels (B)

There was no significant correlation between vitamin D and calcium (r = 0.1667, p = 0.2029) or between vitamin D and uric acid (r = 0.00527, p = 0.97) (Figure 3).

Correlation between serum vitamin D level and lipid profiles

**Figure 4:**
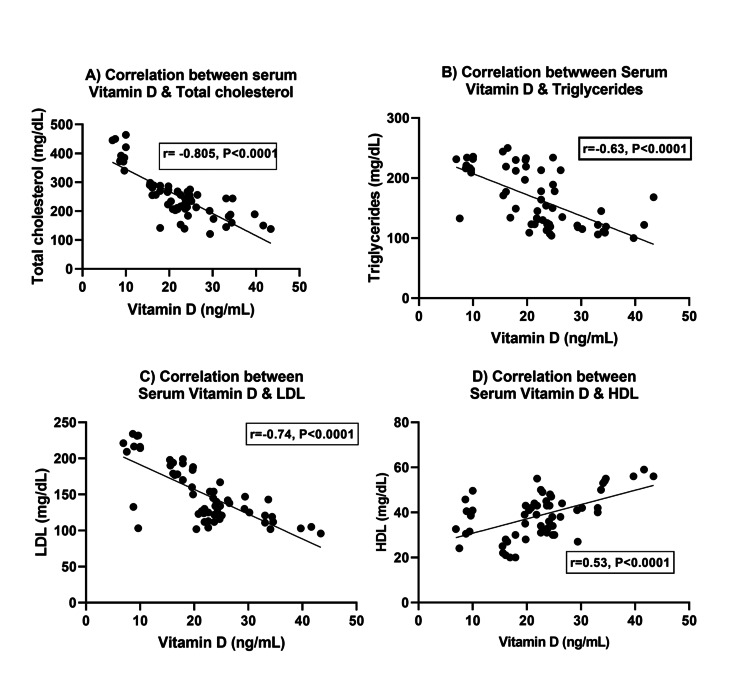
Vitamin D levels and lipid profile: exploring associations with (A) cholesterol, (B) triglycerides, (C) low-density lipoprotein, and (D) high-density lipoprotein

The correlation analysis revealed highly significant correlations between vitamin D levels and lipid parameters. A negative correlation was observed between decreasing levels of vitamin D and cholesterol levels (r = -0.8047, p<0.0001) and triglyceride levels (r = -0.622, p<0.001). Furthermore, a significant inverse relationship was observed between vitamin D and LDL (r = -0.7393, p<0.0001); these findings indicate that lower levels of vitamin D are associated with higher levels of LDL cholesterol. Conversely, there was a significant positive relationship between serum vitamin D and HDL (r = 0.5338, p<0.0001); these results suggest that lower vitamin D levels are associated with lower levels of HDL cholesterol (Figure 4).

Correlation between vitamin D levels and inflammatory factors (CRP and ESR)

**Figure 5:**
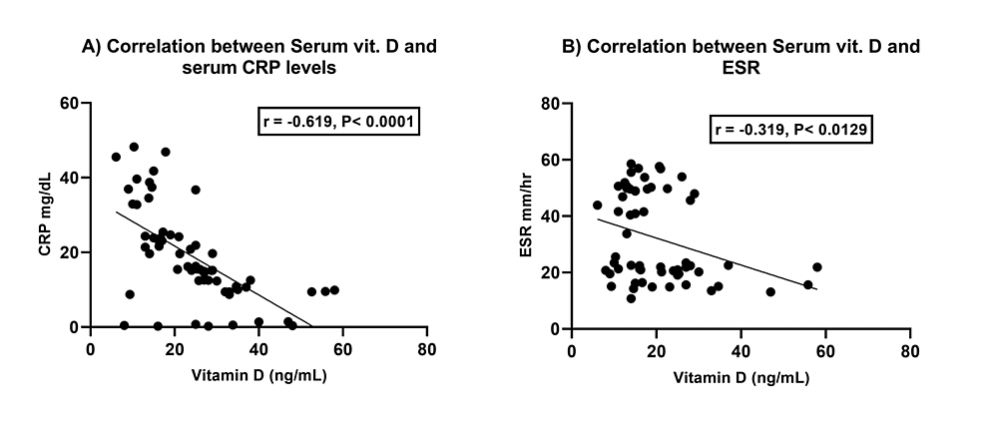
Relationship between vitamin D status and inflammatory markers - (A) C-reactive protein and (B) erythrocyte sedimentation rate

The correlation analysis revealed strong associations between vitamin D status and CRP (p<0.0001) as well as ESR (p<0.0129) levels. The regression coefficients of -0.619 and -0.319, respectively, indicated that as vitamin D levels decrease, there is an increase in both CRP and ESR levels. These findings suggest a potential connection between low vitamin D status and elevated levels of CRP and ESR, highlighting the importance of maintaining adequate vitamin D levels for overall health (Figure 5).

GDF-5 gene polymorphism

We analyzed the prevalence of the GDF-5 T>C (rs143383) polymorphism in all the participants (T represents thymine and C represents cytosine). Our study showed that patients with KOA had a higher TT (homozygous) and TC (heterozygous) genotype prevalence than the control group. The difference between TT and CC was statistically significant (χ2 = 3.86, p = 0.049); in addition, the difference between TC and CC ( χ2 = 5.44, p = 0.019) was statistically significant (Table 2). The TC genotype was found to be a potential risk factor for developing KOA. Based on our findings regarding the frequency of GDF-5 gene alleles, it was observed that the T allele occurred more frequently in individuals with KOA, accounting for 55% of the cases. Conversely, the C allele was more prevalent in the control group, comprising 61% of the cases. This disparity in allele frequencies between the two groups was statistically significant (p = 0.014). These results provide compelling evidence for an association between the GDF-5 gene polymorphism and the development of KOA.

**Table 2:** Comparison of GDF-5 genotypes and allele frequencies between KOA patients and controls GDF-5: growth/differentiation factor 5; KOA: knee osteoarthritis

GDF-5 genotypes	KOA patients		Controls		Chi-square (χ^2^)	P-value	Odds ratio (95% confidence interval)
	N	%	N	%			
TT	18	30	13	22	3.86	0.049	0.33 (0.12-0.89)
TC	30	50	21	35	5.44	0.019	0.32 (0.13-0.78)
CC	12	20	26	43	Reference		
Allele frequencies							
T	66	55	47	39	6.037	0.014	
C	54	45	73	61			

Genotype distribution of GDF-5 polymorphism and its association with vitamin D levels

As shown in Table 3, there was a significant disparity in serum vitamin D levels between KOA patients and controls. Those with varying genotypes (TT, TC, and CC) in the KOA group displayed lower average serum vitamin D levels compared to the control group. These results imply a plausible correlation between genetic variances and vitamin D levels in KOA, which suggests that vitamin D may play a role in the development of this condition.

**Table 3:** Association between GDF-5 genotypes and vitamin D levels in KOA patients GDF-5: growth/differentiation factor 5; KOA: knee osteoarthritis

Genotype group	N	KOA patients	Controls	t-test	P-value
		Vitamin D (mean ± SD)			
TT	31	17.67 ± 7.767	37.53 ± 4.726	8.1698	<0.0001
TC	51	20.64 ± 11.08	37.90 ± 6.22	6.4503	<0.0001
CC	38	22.6 ± 13.05	38.87 ± 6.153	5.2685	<0.0001

## Discussion

KOA is a chronic degenerative joint condition involving the slow deterioration of cartilage, synovial cartilage inflammation, bone outgrowths (osteophytes), and enlargement of subchondral bone [21]. Lack of vitamin D may play a role in the development and exacerbation of KOA. The relationship between reduced serum vitamin D levels and KOA is unclear due to conflicting study results [22].

Our aim in this study was to evaluate the GDF-5 gene polymorphism among obese Saudi patients with KOA. Furthermore, we planned to explore the connection between vitamin D levels with GDF-5 gene polymorphism in obese KOA patients. Our research has shown a significant increase (p<0.0001) in the BMI of patients with KOA when compared to those in the control group. Research has shown that engaging in regular physical activity can lead to a decrease in BMI and help to prevent the negative effects of KOA [23]. Research has also indicated that general and central obesity are connected to a higher chance of developing KOA. Additionally, changes in obesity status have been proven to impact the likelihood of developing KOA [24]. Another study revealed a correlation between obesity and the exacerbation of osteoarthritis, indicating that patients with higher body weight tend to exhibit a more severe clinical course of the condition [25].

We found that patients with KOA have significantly lower levels of vitamin D (p<0.0001) compared to healthy individuals, which is consistent with the findings of Tripathy et al. [26]. Our research showed that patients with KOA had a lower level of GDF-5 protein in their serum due to a GDF-5 gene polymorphism. Furthermore, we found a strong and positive correlation between vitamin D levels and the GDF-5 protein in KOA patients. This is the first study to investigate the relationship between these two variables. This relationship could play a crucial role in the pathogenesis of KOA.

We have observed that various laboratory parameters, including calcium, uric acid, and lipid profiles, showed statistically significant differences between KOA and controls. There was a significant decrease (p<0.0001) in the level of calcium found in the serum of KOA patients compared to the control group. New research has revealed the impact of calcium on chondrocytes, specifically on matrix protein synthesis, cytoskeleton remodeling, apoptosis, and the regulation of proteoglycans. The study found that lower levels of serum calcium were associated with more severe KOA and that calcium may even have a protective effect [27].

Our study has revealed a significant increase in the levels of serum uric acid in patients with KOA when compared to the control group (p<0.001). This disparity emphasizes the potential role of uric acid in the development of KOA and provides valuable insights into its relevance for understanding the underlying mechanisms of the disease. A study by Bassiouni et al. (2021) reported that there is a strong link between uric acid levels and the severity of KOA in female patients. Interestingly, this connection was not observed in male patients. These results highlight the importance of understanding the gender-specific nature of the relationship between uric acid and KOA severity [28].

Our research yielded compelling evidence of a significant rise in lipid profiles, encompassing cholesterol, triglyceride, LDL-C, and a decrease in HDL-C levels, among individuals with KOA compared to their healthy counterparts (p<0.0001). These observed differences bear statistical significance, reinforcing the notion that lipid abnormalities are intricately linked to the development and progression of KOA. A study by Zhou et al. (2017) found a strong link between blood lipid abnormalities and a higher risk of knee pain and clinical KOA in middle-aged and older adults [29].

The current study found that ESR and CRP levels in KOA patients were significantly higher (p<0.0001) than in controls. According to a study by Tetik et al., samples taken from patients who have both rheumatoid arthritis (RA) and OA revealed significantly higher levels of CRP and ESR compared to the control group. These results demonstrate the existence of systemic inflammation in both RA and OA. These inflammatory markers may help to evaluate disease activity and track the development of these joint disorders [30]. A different study reported that individuals who suffer from KOA and experience swelling and tenderness symptoms typically have increased ESR and CRP levels [31].

It has been found that there is a strong negative correlation between vitamin D levels and BMI, which supports the findings of Kumaratne et al. [32]. According to Tripathy et al., BMI and serum vitamin D levels are also linked to KOA. Specifically, a higher BMI is associated with lower vitamin D levels. However, there was no significant correlation between calcium and uric acid levels similar to our study [26]. Furthermore, Saadat et al. discovered no statistically significant association between uric acid and vitamin D [33]. Our findings contrast with those by Isnuwardana et al. [34], which suggested an inverse relationship between vitamin D deficiency and hyperuricemia.

Our research focused on the impact of vitamin D levels on cholesterol, triglycerides, LDL-C, and HDL-C. We discovered a significant negative correlation between vitamin D and cholesterol, triglycerides, and LDL-C. However, we found a positive relationship between vitamin D and HDL-C. The study by Kim and Jeong also confirmed a significant correlation between vitamin D levels and lipid profiles [35]. Our findings indicate that vitamin D has an impact on lipid metabolism. This is consistent with previous studies among the adult population, such as the research by Wang et al., which investigated the impact of serum vitamin D on lipids in Chinese adults. The study found a significant correlation between serum vitamin D levels and lipid parameters. These results emphasize the importance of maintaining adequate vitamin D levels for lipid regulation across different age groups and populations [36].

Our study noted the reciprocal relationship between vitamin D levels and inflammatory markers. We observed a significant inverse correlation between vitamin D and two key inflammatory factors, ESR and CRP. These findings imply that lower levels of vitamin D are associated with higher levels of ESR and CRP, indicating a potential role for vitamin D in modulating the inflammatory response. Amirkhizi et al.'s study found that people with KOA had lower vitamin D levels and higher CRP and ESR levels than healthy individuals (p<0.0001) [11].

Our study found a noteworthy correlation between the GDF-5 T>C (rs143383) polymorphism and KOA. Additionally, there was a significant difference in GDF-5 allele frequencies between KOA patients and controls. Specifically, the TC genotype polymorphism was more common among KOA patients than in the control population. These results emphasize the possible involvement of the GDF-5 T>C (rs143383) polymorphism as a factor in the development and vulnerability of KOA. Furthermore, the T allele was frequently observed in individuals with KOA. Several studies have found a significant link between the GDF-5 gene polymorphism and KOA [37-40]. Additionally, Pan et al. discovered that the GDF-5 gene is a susceptibility gene for osteoarthritis, and its down-regulation plays a role in developing this condition. These studies suggest that the GDF-5 T>C (rs143383) polymorphism is a crucial factor in the progression of OA and sheds light on the involved molecular mechanisms [40]. On the other hand, a Genome-Wide Association Study (GWAS) performed among a Japanese population discovered three new risk loci and a possible candidate gene for KOA [41]. These findings suggest that the genetic factors that contribute to KOA are quite complex, and more research is necessary to determine the underlying genetics of this condition. It is important to note that not all studies have found a significant correlation between the GDF-5 gene polymorphism and KOA. However, it is remarkable that in the study conducted by Mohasseb et al., no significant association was observed between the GDF-5 polymorphism and susceptibility to KOA in the study population. The authors acknowledged that their findings were inconsistent with previous research demonstrating a significant link between the GDF-5 polymorphism and KOA. They postulated that discrepancies in study design, sample size, and genetic background might have contributed to these disparities [42].

The connection between genetic and environmental factors in OA is complex, leading to variations in study results. Genetic predisposition and environmental influences both play a role in OA development. Differences in genetic profiles among populations studied and variations in study design can also contribute to inconsistent findings [43]. Understanding these factors is crucial for a complete understanding of OA's genetic landscape. In addition to what was previously mentioned, there is evidence to suggest that another GDF-5 polymorphism in the same area, called rs143384, may impact the expression of GDF-5 T>C (rs143383). This interaction between different GDF-5 gene polymorphisms can affect how GDF-5 is expressed [44].

To the best of our knowledge, no previous research has investigated the correlation between the GDF-5 genotype and its protein and vitamin D levels in KOA patients, specifically within Saudi Arabia. Our study aimed to address this gap, and we discovered that GDF-5 (rs143383) polymorphism is linked to low GDF-5 protein expression. Furthermore, our results revealed a positive significant correlation between vitamin D and GDF-5 protein and the association between vitamin D deficiency and GDF-5 polymorphism. This may contribute to the pathogenesis and progression of KOA.

Limitations of the study

This study has certain limitations that warrant acknowledgment. Firstly, the sample size of 60 KOA patients is relatively small, which constrains the generalizability of the findings. Additionally, the study's focus on obese female patients with KOA in Saudi Arabia limits its applicability to other populations. Differences in patient selection and genotyping methods can cause inconsistencies in genetic association studies in OA. Moreover, the lack of a functional analysis and the reliance on self-reported data may introduce further constraints. Consequently, it is imperative to validate these findings by conducting larger-scale studies involving non-obese individuals and male patients.

## Conclusions

Our findings revealed a significant relationship between the GDF-5 T>C (rs143383) gene polymorphism, a reduction in GDF-5 protein, and vitamin D deficiency in obese Saudi women with KOA. This discovery suggests that genetics may play a crucial role in the development of KOA and a possible link between a decrease in GDF-5 and vitamin D deficiency in its pathophysiology. In addition, the TC genotype might increase the risk of KOA. These findings offer valuable insights into the complex interplay of genetics, vitamin D, and KOA and provide helpful information for further research and management strategies.
